# Medication Errors in Vietnamese Hospitals: Prevalence, Potential Outcome and Associated Factors

**DOI:** 10.1371/journal.pone.0138284

**Published:** 2015-09-18

**Authors:** Huong-Thao Nguyen, Tuan-Dung Nguyen, Edwin R. van den Heuvel, Flora M. Haaijer-Ruskamp, Katja Taxis

**Affiliations:** 1 Department of Clinical Pharmacy, School of Pharmacy, University of Medicine and Pharmacy at Ho Chi Minh city, Ho Chi Minh, Vietnam; 2 Department of Epidemiology, University Medical Center Groningen, University of Groningen, Groningen, the Netherlands; 3 Department of Mathematics and Computer Science, Eindhoven University of Technology, Eindhoven, the Netherlands; 4 Department of Clinical Pharmacology, University Medical Center Groningen, University of Groningen, Groningen, the Netherlands; 5 Department of Pharmacy, Unit of Pharmacotherapy and Pharmaceutical Care, University of Groningen, Groningen, the Netherlands; Queen's University Belfast, UNITED KINGDOM

## Abstract

**Background:**

Evidence from developed countries showed that medication errors are common and harmful. Little is known about medication errors in resource-restricted settings, including Vietnam.

**Objectives:**

To determine the prevalence and potential clinical outcome of medication preparation and administration errors, and to identify factors associated with errors.

**Methods:**

This was a prospective study conducted on six wards in two urban public hospitals in Vietnam. Data of preparation and administration errors of oral and intravenous medications was collected by direct observation, 12 hours per day on 7 consecutive days, on each ward. Multivariable logistic regression was applied to identify factors contributing to errors.

**Results:**

In total, 2060 out of 5271 doses had at least one error. The error rate was 39.1% (95% confidence interval 37.8%- 40.4%). Experts judged potential clinical outcomes as minor, moderate, and severe in 72 (1.4%), 1806 (34.2%) and 182 (3.5%) doses. Factors associated with errors were drug characteristics (administration route, complexity of preparation, drug class; all p values < 0.001), and administration time (drug round, p = 0.023; day of the week, p = 0.024). Several interactions between these factors were also significant. Nurse experience was not significant. Higher error rates were observed for intravenous medications involving complex preparation procedures and for anti-infective drugs. Slightly lower medication error rates were observed during afternoon rounds compared to other rounds.

**Conclusions:**

Potentially clinically relevant errors occurred in more than a third of all medications in this large study conducted in a resource-restricted setting. Educational interventions, focusing on intravenous medications with complex preparation procedure, particularly antibiotics, are likely to improve patient safety.

## Introduction

Medication errors are a global issue, especially prescribing and administration errors [1,2]. Drug administration is important because the possibilities to prevent or correct errors at this stage are limited. Two recent systematic reviews show median error rates between 8% and 10% (excluding time errors) in medication preparation and administration [3,4]. Most of these studies have been carried out in developed countries. In such countries, patient safety issues have been recognised a long time ago and efforts to increase medication safety such as implementation of electronic prescribing systems, barcoding, and involving clinical pharmacists at the ward level are on-going [5,6].

Little is known about patient safety in resource-restricted settings, i.e. developing and transitional countries [7]. One of the few large studies found that 2.5% to 18.4% of hospital admissions were associated with an adverse event and about 30% of those resulted in the death of the patient which was much higher than those in developed countries [8]. Poor health system infrastructure and inadequately trained healthcare staff probably contributed to this [7,8]. Two small scale studies on medication errors, each including about one thousand preparations and administrations, have been carried out in a Malaysian teaching hospital [9,10]. One study was conducted on two paediatric wards [9] and the other was done on a haematology ward [10]. Medications were supplied to wards either as a bulk for the whole ward (for commonly used medications) or as unit-doses (for more specific medications). There were no clinical pharmacy services at ward level. Error rates were around 8%, which is comparable to the median error rate reported by Keers *et al*. [3]. More evidence is needed from other resource-restricted settings. Especially, studies with a larger sample size which allow investigation of factors contributing to errors to identify appropriate approaches to prevent errors. In this study, we determined the prevalence and potential clinical outcome of medication preparation and administration errors in two Vietnamese hospitals and identified associated factors in a multifactorial model.

## Methods

### Study design and setting

This prospective study, using an observation-based approach, took place on six wards in two major public hospitals in a large city in Vietnam. Both were provincial general hospitals, hospital A had 700 beds and hospital B had 1000 beds. In each hospital we studied an intensive care unit (ICU, 20 beds each ward) and a post-surgery ward (PS, 12 beds each ward), in hospital A we also studied one general internal medicine ward (GIM, 56 beds) and in hospital B one trauma unit (TU, 48 beds). The number of nurses varied across the wards, but in general, in each shift, each nurse was assigned three to six patients. Most nurses held an Associate’s Degree in Nursing.

Commonly used medications were kept at the ward. The remainder of the medications was dispensed from the pharmacy department every morning for the weekdays, and on Friday morning additionally for the weekend. Orders for medications were written by doctors onto the patient’s medical records. Nurses transcribed prescriptions either manually into inpatient drug charts (paper, hospital A) or entered them into the patients’ electronic drug use records in a computer and printed out the drug regimen for each patient (hospital B). All medications were prepared and administered by nurses referring to these charts. Medication preparations were undertaken either in a separate room or on a dedicated trolley. Every drug administration on the ward was recorded in the nurse chart and on a disclosure form at the patient’s bedside which was attached to the patient’s medical record on discharge. Clinical pharmacists were not available at ward level.

### Data collection

Data were collected between March and June 2011 by four pharmacy students (two in each hospital) using direct observation [11,12]. This study was part of a larger project and results on errors related to insulin have already been published [12]. More details on data collection methods can be found there. The students were trained for about a week in observation technique by lectures on medication error research as well as ward-based observations by a senior researcher to ensure all observers used the same definition of an error. A one-day observation pilot was conducted on each study ward prior to commencement of the main study to help the observers get familiar with medications and procedures of the study wards. This also helped nursing staff get comfortable with someone being around and minimized the Hawthorne effect [13].

At the beginning of each drug round, the observer asked the nurse in charge for permission to observe. Nurses were told that the observer was a pharmacy student who wanted to learn more about ward-based drug preparation and administration. The observer followed the nurses during all intravenous and oral drug preparation and administration. Data were collected 12 hours per day (7am-7pm) on seven consecutive days (Monday–Sunday) on each ward. Two observers were present at the same time to maximise the number of observations per drug round in case several nurses worked in parallel. Details of drug preparation and administration were recorded on the pre-specified data collection forms. Nurses’ and patients’ identity was anonymised and kept confidential. For ethical reasons, the observer intervened in case of potentially serious medication errors about to reach the patient in a non-judgmental manner. These errors were also included in the analysis. After each round of observation, the observer went through all the observation notes and compared the information observed with the doctor’s orders to detect any discrepancies. All collected data was revised by a senior researcher to ensure the validity and reliability of the data. Disagreements were resolved through discussion.

### Ethical consideration

The study was approved by Medical Ethics Committee and Management Board of the study hospitals (Trung Vuong Emergency hospital and Gia Dinh General hospital, Ho Chi Minh city, Vietnam). As highlighted above, nurses provided verbal consent to participate in the study. This was documented on the observation sheets. The research involved minimal risk for the nurses (data were anonymized) therefore written consent was not obtained to minimise administrative load. This procedure was approved by both Medical Ethics Committees.

### Definitions

Medication errors were defined as deviations in preparation and administration of oral or intravenous medications from the doctors’ prescriptions, the hospital policies and procedures or the manufacturers’ instructions [10,14]. Medication errors were classified into the following categories, similar to that used by other authors [15,16]: wrong drug, wrong dose, wrong dosage-form, deteriorated drug, wrong preparation technique, omission, unordered drug, and wrong administration technique. Doses given earlier or later than the prescribed time were not counted as errors. An error could be classified in one category only (Table 1).

**Table 1:** Types of medication errors.

Type of errors	Definition
**Preparation**	
Wrong drug	Preparation of a drug which differs from that prescribed
Wrong dose	Preparation of a dose that is higher than, or less than, the amount prescribed (± 10%)
Wrong dosage-form	Formulation of drug deviates from that prescribed
Deteriorated drug	Preparation of a drug that has expired or for which the physical or chemical dosage-form integrity has been compromised
Wrong preparation technique	Inappropriate procedure or improper technique in the preparation of a drug (compared to the manufacturer’s instructions or hospital policy, including wrong diluent, wrong solvent, wrong volume, possible incompatibility)
**Administration**	
Omission	Failure to administer an ordered dose to a patient
Unordered drug	Administration to the patient of non-prescribed medication
Wrong administration technique	Inappropriate procedure or improper technique in the administration of a drug (rate, incompatibility, route, dose (± 10%) if prepared with correct dose). A rate error was identified if administration took less than 3 or more than 5 min (for a bolus dose) or 15% shorter/longer than the required infusion time (for an infusion dose). An incompatibility error was determined if there was incompatibility information available in at least one of four documents including the *Handbook on Injectable Drugs*, *15* ^*th*^ *edition* [17], *AHFS Drug Information 2009* [18], Vietnam National Drug Formulary, 2nd edition [19] and the manufacturers’ instructions.

### Potential clinical outcomes of errors

Four healthcare professionals (one doctor, one nurse, and two pharmacists) scored the potential clinical outcome of each medication with one or more errors (i.e. erroneous dose). A 10 point scale from zero (labelled as *no harm*) to 10 (*death*) was used. We calculated the mean score. A value below 3 suggested a minor outcome, of 3–7 a moderate outcome, and above 7 a severe outcome. This has been shown to be a valid and reliable method [20].

### Data analysis

#### Calculation of error rate

We calculated the overall error rate (with 95% confidence interval) as percentage by dividing the number of doses with one or more errors (i.e., erroneous doses) by the sum of given doses plus omitted doses, which were called total opportunities for errors (TOEs), then multiplying it by 100 (Eq 1). Likewise, the rate of each error type was calculated by dividing the number of errors of that particular type by the sum of given doses plus omitted doses, then multiplying it by 100.

$$Overall\;error\;rate=\;\frac{The\;number\;of\;doses\;with\;one\;or\;more\;errors}{Given\;doses+omitted\;doses\;\left(TOEs\right)}\;x\;100$$ (1)

**Eq 1. Overall error rate calculation (TOEs = total opportunities for errors)**

#### Multivariable logistic regression

Multivariable logistic regression was performed to explore factors associated with errors. Independent variables were characteristics of drug (ATC—Anatomical Therapeutic Chemical—class, complexity of preparation, and administration route), administration time (day of the week, drug round) and experience of nurse in charge, corrected for hospital, ward, and observer. Data were analyzed using SPSS statistical package (SPSS 20.0, SPSS Inc., IBM Corporation, Somers, NY, USA).

#### Definition of variables

The following definitions were used: medications were grouped using the ATC/WHO classification (http://www.whocc.no/atc_ddd_index), less commonly used medications, i.e. frequency around 5% or lower, were grouped as others. A medication preparation was defined as follows: simple preparations did not involve any manipulations, e.g. this included drawing up an injectable solution with a syringe. Complex oral preparations included manipulations such as crushing tablets or opening capsules and dissolving them. Complex intravenous preparations included manipulations of one or more steps such as reconstituting a medication. Administration route were either intravenous (including short IV injections, bolus, and infusions) or oral. According to study wards/hospitals, there were five drug rounds a day: morning (7am-11am), lunch (11am-2pm), afternoon (2pm-5pm), evening (5pm-9pm), and night (after 9pm till 7am next day). So, our observation time (7am to 7pm) included four drug rounds. Nurse experience was classified into four groups: ≤ 1, >1–2, >2–6, and > 6 working years. We also included interactions terms (i.e. the effect of a specific factor was modified by the others) into the model. Backward elimination using the likelihood ratio test was applied to test for the effects of interactions and variables. The level of significance was set at 0.05.

## Results

A total of 6232 medications were prescribed during the study period. Among those, 5271 (84.6%) drug preparations and administrations involving 327 patients were included: 2996 in hospital A and 2275 in hospital B.

### Prevalence and potential clinical outcomes of errors

In total, 2060 out of 5271 doses had at least one error, affecting 92.4% (302 out of 327) patients. The error rate was 39.1% (95% confidence interval (CI) 37.8%- 40.4%). Among those, 336 doses had 2 errors and 8 had 3 errors. Overall, 2412 errors were identified. Experts judged potential clinical outcomes as minor, moderate, and severe in 72 (1.4%), 1806 (34.2%) and 182 (3.5%) doses.

Most frequent errors were wrong administration technique (23.5%), followed by wrong preparation technique, omission, and wrong dose (15.7%, 2.3%, and 1.8%, respectively). There were no wrong dosage-form errors (Table 2).

**Table 2:** Frequencies of error types (n = 2412 errors out of 2060 erroneous doses).

	Hospital A			Hospital B			Total	Rate (%)
Error type	ICU	PS	GIM	ICU	PS	TU		
Wrong drug	2	0	55	3	2	2	64	1.2
Wrong dose	8	7	10	26	24	18	93	1.8
Wrong dosage-form	0	0	0	0	0	0	0	0.0
Deteriorated drug	5	0	0	0	4	0	9	0.2
Wrong preparation technique	316	40	88	104	201	81	830	15.7
Omission	2	1	60	21	0	39	123	2.3
Unordered drug	1	8	12	7	13	15	56	1.1
Wrong administration technique	257	212	191	136	265	176	1237	23.5
Total	591	268	416	297	509	331	2412	

ICU: intensive care unit, PS: post-surgery, GIM: general internal medicine, TU: trauma unit.

The observers intervened twice to prevent errors from occurring: one involved a mixture of 10 IU fast-acting insulin and 10 mL KCl 10% in glucose 5% to the wrong patient, the other was the wrong dose of midazolam (50 mg instead of 25 mg) which was about to be added to an infusion bag.

### Factors associated with errors

Using backward elimination of interactions and variables in the logistic regression analysis, corrected for hospital, ward, and observer, we observed that errors were associated with characteristics of the drug (administration route, complexity of preparation, ATC class, all p values < 0.001), and administration time (drug round, p = 0.023; day of the week, p = 0.024). All two-way interactions between administration route, complexity of preparation, and ATC class were significant (all p values < 0.001). There was also a significant interaction between ATC class and drug round (p = 0.007). Nurse’s experience was not significant.

To give more insight into the data, Tables 3–5 describe error rates stratified by significant factors and interactions.

**Table 3:** Number of included doses (n) and error rates (%) stratified by drug class, administration route and preparation procedure.

Administration route	Oral				Intravenous				Total	
Preparation procedure	Simple		Complex		Simple		Complex			
	*n*	*%*	*n*	*%*	*n*	*%*	*n*	*%*	*n*	*%*
**ATC class**										
A	731	9.4	370	21.1	72	91.7	187	92.5	1360	28.4
B	106	17.0	97	7.2	531	33.0	101	77.2	835	33.3
C	418	10.0	188	18.1	66	84.8	71	98.6	743	27.2
J	59	10.2	2	0.0	170	63.5	724	89.2	955	79.6
N	437	5.5	84	11.9	180	63.9	70	95.7	771	28.0
Others	255	20.4	182	3.3	79	91.1	91	96.7	607	35.9
Total	2006	10.5	923	14.6	1098	53.9	1244	90.2	5271	39.1

ATC: Anatomical Therapeutic Chemical

**Table 4:** Number of included doses (n) and error rates (%) stratified by day of the week, administration route and preparation procedure.

Administration route	Oral				Intravenous				Total	
Preparation procedure	Simple		Complex		Simple		Complex			
	*n*	*%*	*n*	*%*	*n*	*%*	*n*	*%*	*n*	*%*
**Day of the week**										
Sunday	271	7.0	124	15.3	147	53.1	201	91.5	743	40.4
Monday	317	13.2	54	24.1	128	56.3	133	93.2	632	39.7
Tuesday	276	10.1	134	11.9	147	48.3	162	88.9	719	36.0
Wednesday	252	11.1	176	15.9	182	56.6	195	91.8	805	42.0
Thursday	314	10.2	165	10.9	181	51.9	190	86.8	850	36.4
Friday	298	10.7	139	13.7	167	54.5	169	87.6	773	37.5
Saturday	278	10.8	131	16.8	146	56.8	194	91.8	749	41.8
Total	2006	10.5	923	14.6	1098	53.9	1244	90.2	5271	39.1

**Table 5:** Number of included doses (n) and error rates (%) stratified by drug class and drug round.

Drug round	Morning		Lunch		Afternoon		Evening		Total	
	*n*	*%*	*n*	*%*	*n*	*%*	*n*	*%*	*n*	*%*
**ATC class**										
A	618	32.2	253	31.6	404	18.6	85	37.6	1360	28.4
B	354	34.5	207	40.1	212	25.9	62	29.0	835	33.3
C	308	26.6	144	29.2	232	26.3	59	28.8	743	27.2
J	482	80.1	203	80.8	199	80.9	71	69.0	955	79.6
N	269	22.7	168	36.3	231	29.0	103	26.2	771	28.0
Others	248	39.1	111	28.8	190	31.6	58	50.0	607	35.9
Total	2279	41.6	1086	42.5	1468	32.6	438	39.3	5271	39.1

ATC: Anatomical Therapeutic Chemical

Much higher error rates were observed for intravenous medications than for oral ones (73.2% vs. 11.8%), and for complex preparation procedures than for simple ones (58.0% vs. 25.9%). Higher error rates were observed for intravenous medications involving complex preparation procedures than for simple intravenous ones (90.2% vs. 53.9%). This was consistent for most drug classes.

In terms of drug class, the highest error rates (79.6%) were observed for anti-infective drugs (ATC class J). Low error rates were observed for cardiovascular drugs (ATC class C), but again, high error rates were observed for complex preparation procedures and intravenous administrations of cardiovascular medications (Table 3).

Medication errors seemed to be similar for all days of the week with error rates ranging from 36.0% to 42.0%. When stratifying the data by administration route and preparation procedure, almost all error rates were higher on Mondays compared to other days (Table 4).

Slightly fewer errors occurred during afternoon rounds than at other times of the day. This was not consistent across all drug classes. For example, error rates of anti-infective medications (ATC class J) were similar across drug rounds (Table 5).

## Discussions

Our study on six wards in two Vietnamese hospitals showed that in more than a third of all medication preparations and administrations potentially clinically relevant errors occurred. Errors were associated with drug characteristics (intravenous medications, complex preparation procedures, anti-infectives), and administration time (drug round and day of the week), but not associated with nursing experience.

The error rate of 39.1% (37.8%- 40.4%) identified in our study is relatively high. Previous studies using similar methodology including oral and injectable medications found error rates (without time errors) between 7.5% and 33% [10,16,21,22]. Our higher error rate may be partly due to observing a high proportion of intravenous medications. This was 44% in our study whereas between 9% [21] and 26% [10] intravenous/injectables were observed in other studies. In addition, the other studies did not include all days of the week [10,16,21,22]. Furthermore, there may have been subtle differences in the definition of a medication error. We were strict about criteria of preparation technique errors, where mixing/shaking errors were included (for example a dose of ceftriaxone 1g was incompletely dissolved in 10 mL sterile water because of insufficient shaking) while other authors did not clearly specify this. In contrast to the previous studies, the majority of erroneous doses in our study were judged to be potentially clinically significant and only few were considered minor. However due to different methods used to assess clinical outcome these data are difficult to compare.

In line with a review on intravenous medication errors [23] we observed frequently administration technique, preparation technique, omission and dose errors. About half of errors were wrong administration technique and most of these were rate errors involving bolus medications, which should have been given within 3–5 minutes according to the Vietnam National Drug Formulary [19]. The second most common error involved preparation technique where nurses did not shake or mix properly while reconstituting intravenous medications or used the wrong volume of solvent/diluent. For oral medications, the most frequent preparation technique errors were crushing tablets or capsules that should not have been crushed, for example, sustained or extended release dosage-forms or coated tablets. Most omission and dose errors involved oral medications which were unavailable or where nurses misinterpreted/mistranscribed the prescriptions. In around 40% of dose errors, half the dose or twice the dose was given. For example, two tablets of alpha chymotrypsin were given in the morning instead of administering this in two doses (morning and evening) as the doctor intended. This was probably due to ambiguous prescriptions: alpha chymotrypsin 1 tablet x 2. We did not find this type of error being reported in other studies.

Intravenous administration route had a bigger effect on error occurrence compared to oral one. The potential for errors increased when the (intravenous) medication involved a complex preparation process. This was in line with a previous study on intravenous medications showing that multiple step preparations involved more errors than simple ones [24]. A review estimated that removing the reconstitution step (complex preparations) by providing prepared injections (simple preparations) would reduce the overall error rate from 73% to 17% [23]. Anti-infective drugs for systemic use were shown as the most error-prone medications as most antibiotics observed involved complex preparation procedures (724 out of 955, Table 3). We also found an association between administration time and errors. The risk for errors was higher at all times during the day except the afternoon round. A reason for this may be that the afternoon was the least busy time of the day for the nurses. For instances, in the morning nurses also had to take blood samples, transcribe prescriptions and during lunch and evening rounds, they had to give out meals. Underlying this could be frequent interruptions of staff during busy times on the wards [25,26] or the number of patients a nurse has to take care of [16,21]. Day of the week was a significant term which remained in the final logistic regression model, but it was not easy to identify the most risky day for errors. This is probably due to unequal distribution of medications observed in terms of drug characteristics (i.e. administration route, preparation procedure, and ATC class) across days of the week. For example, a higher proportion of simple oral medications was prepared and administered on Monday (50.2%) compared to other days. When administration route and preparation procedure were kept constant, Monday turned out to be an error-prone day. This has been reported in another study [22].

Nursing experience was not significant, irrespective of administration route as well as type of preparation. In contrast, an Australian study on intravenous medications found that each year of experience, up to 6 years, reduced the risk of error by 11% [27]. Maybe in Australia, nurses are continuously trained after graduation, while in Vietnam continuing education for healthcare professionals, including nurses, is a concept introduced recently [28]. Nurses may have learnt from the senior ones and they have little opportunities to update knowledge and practice. Alternatively, senior nurses may be better than junior ones, but because of more responsibilities they may have to carry out several tasks simultaneously and may be interrupted more often which is associated with errors [25,26].

A range of different measures are recommended for error reduction including improving the competence of healthcare professionals, controlling working environment, and enhancing the safety culture [6,29]. Preparation technique errors could be reduced by providing medications, particularly intravenous ones, with simple preparations such as ready to use medications which either prepared by pharmaceutical company or pharmacy department [23]. Administration technique errors, especially rate errors, could be eliminated by using (smart) infusion pumps [30]. However, in the context of resource limited settings, the implementations of such interventions may be too costly and unfeasible. Nurse experience had no impact on the error rate. Guidelines seemed to be absent or not up to date on the study wards. This would suggest starting improvements by introducing an educational training programme for nurses targeting the most error-prone medications (intravenous medication involving complex preparation procedures) as well as providing guidelines. Recent studies confirmed that education, protocols and guidelines are a successful approach to reduce medication error rates, also in resource-restricted settings [31–33]. This would be in line with patient safety research from resource restricted settings identifying inadequate training of clinical staff and lack of protocol/policy as important factors [7,8]. Errors were likely to occur during busy drug rounds (all drug rounds except afternoon). This would suggest that working patterns and/or system of medication management should be adapted to assign comparable workload for every drug round [31], and to minimize interruptions [26,34–36], or to have dedicated nurses who are responsible for medication administrations, but research on the latter is inconclusive so far [37]. As a first step to foster a culture of safety, clinical/hospital pharmacists could discuss the errors and prevention strategies with ward staff. This would stimulate interests and concerns in patient safety, and create a comfortable environment to learn from errors. In later stages, implementation of an (non-punitive) error reporting system would be recommended. In spite of underestimation, this system has been indicated as a valuable tool for preventing future errors [38].

### Strengths and limitations

In contrast to most previous studies on administration errors, we studied a large number of variables and interactions between these variables as potential factors contributing to errors. For instance, Chua *et al*. used χ^2^ test to find the effect of administration route and drug round [10]. But an imbalance, e.g. more intravenous medications given during a particular round, will not be taken into account in univariable analysis. For example our descriptive data showed no differences in error rates between days of the week. Only after stratification, we found higher error rates on Mondays. Surprisingly, we found a number of significant interactions. This suggested that the complexity of clinical practice should be taken into account and confirmed the necessity of controlling for other factors, known or suspected to have an impact on errors, while evaluating potential factors. These findings have not been reported in the literature.

We used direct observation to detect errors which is recognized as the “gold standard” [11,39]. The observation rate was high (84.6%) and observations were only missed in some cases, for example, if there was more than one nurse preparing/administering drugs at the same time or in cases where observation was inappropriate for seriously ill patients. It should be noted that part of the evening rounds and night shifts were not included. Therefore, the error rate during the night remains unknown. Almost all patients (92.4%) experienced at least one medication error during their hospital stay. As with all observation based studies, we did not collect data on the real consequences of errors, but if only a fraction of the 3.5% potentially severe cases result in actual harm, there would be many patients affected, given the fact that about one thousand medications were administered per week on each ward.

## Conclusions

In this large study of medication preparation and administration errors in a resource-restricted setting, we found that potentially clinically relevant errors occurred in more than a third of all medications. Administration technique, preparation technique, omission, and dose errors were most commonly encountered. Interventions, probably starting with education, focusing on intravenous medications with complex preparation procedure, particularly antibiotics, are needed to improve patient safety.
